# The Complement System in Ovarian Cancer: An Underexplored Old Path

**DOI:** 10.3390/cancers13153806

**Published:** 2021-07-28

**Authors:** Yaiza Senent, Daniel Ajona, Antonio González-Martín, Ruben Pio, Beatriz Tavira

**Affiliations:** 1 Translational Oncology Group, Program in Solid Tumors, Cima University of Navarra, 31008 Pamplona, Spain; ysenent@alumni.unav.es (Y.S.); agonzalezma@unav.es (A.G.-M.); rpio@unav.es (R.P.); btavirai@unav.es (B.T.); 2 Department of Biochemistry and Genetics, School of Sciences, University of Navarra, 31008 Pamplona, Spain; 3 Navarra Institute for Health Research (IdISNA), 31008 Pamplona, Spain; 4 Centro de Investigación Biomédica en Red de Cáncer (CIBERONC), 28029 Madrid, Spain; 5 Department of Oncology, Clinica Universidad de Navarra, 28027 Madrid, Spain; 6 Department of Pathology, Anatomy and Physiology, School of Medicine, University of Navarra, 31008 Pamplona, Spain

**Keywords:** ovarian cancer, adaptive immunity, innate immunity, complement system, immunotherapy, cancer immunology, tumor microenvironment

## Abstract

**Simple Summary:**

Ovarian cancer is one of the leading causes of death among women and the most lethal cause of death from gynecological malignancy in developed countries. The immune system plays an essential role in ovarian cancer progression, and its modulation may be used as an effective therapeutic tool. In this review, we examine the relevance of the cellular and humoral components of the adaptive and innate immune responses in ovarian cancer, focusing on the role of an essential component of innate immunity, the complement system. Elements of this system show tumor-promoting activities that impede the efficacy of developing treatment strategies. We discuss evidence that suggests a role of complement components in the progression of ovarian cancer and provide a rationale for evaluating the inhibition of complement components in combination with immunotherapies aimed to reactivate antitumor T-cell responses.

**Abstract:**

Ovarian cancer is one of the most lethal gynecological cancers. Current therapeutic strategies allow temporary control of the disease, but most patients develop resistance to treatment. Moreover, although successful in a range of solid tumors, immunotherapy has yielded only modest results in ovarian cancer. Emerging evidence underscores the relevance of the components of innate and adaptive immunity in ovarian cancer progression and response to treatment. Particularly, over the last decade, the complement system, a pillar of innate immunity, has emerged as a major regulator of the tumor microenvironment in cancer immunity. Tumor-associated complement activation may support chronic inflammation, promote an immunosuppressive microenvironment, induce angiogenesis, and activate cancer-related signaling pathways. Recent insights suggest an important role of complement effectors, such as C1q or anaphylatoxins C3a and C5a, and their receptors C3aR and C5aR1 in ovarian cancer progression. Nevertheless, the implication of these factors in different clinical contexts is still poorly understood. Detailed knowledge of the interplay between ovarian cancer cells and complement is required to develop new immunotherapy combinations and biomarkers. In this context, we discuss the possibility of targeting complement to overcome some of the hurdles encountered in the treatment of ovarian cancer.

## 1. Current Status of Ovarian Cancer: Clinical Perspective and Needs

Ovarian cancer is the most lethal gynecological cancer in developed countries [1]. According to data from the US National Cancer Institute (NIH), the five-year survival rate for ovarian cancer is 49.1% [2]. This can be attributed to a delay in the diagnosis due to the lack of specific symptoms; 70% of cases are diagnosed in stage III or IV, making it difficult to treat with curative intent [3]. Ovarian cancer is a complex disease that comprises different tumor types, of which epithelial ovarian cancer represents 90–95% of all cases [4]. The current standard treatment includes surgery and platinum-based chemotherapy followed by a maintenance period with the anti-angiogenic therapy bevacizumab [5]. Initial responses to chemotherapy are frequently high, but unfortunately, up to 70% of patients experience recurrence within the first three years, especially patients who are late-diagnosed [5]. Survival rates have recently improved with the introduction of a new generation of poly (ADP-ribose) polymerase inhibitors (PARP inhibitors (PARPi)). These drugs, administered after chemotherapy, prolong the time during which the disease does not progress, mainly in patients carrying BRCA mutations [6]. Despite this great advance, the overall survival of patients with ovarian cancer is still low. There are a variety of factors associated with chemoresistance and relapse, including interactions between ovarian cancer cells and their surrounding immune microenvironment [7]. Ovarian cancers are considered “immunogenic tumors” in which spontaneous antitumor immune responses have been demonstrated [8,9]. The presence of tumors infiltrating CD8^+^ lymphocytes in the tumor microenvironment (TME) is associated with longer recurrence-free and overall survival [10,11], whereas the recruitment of regulatory T (Treg) cells is correlated with a poor outcome [12]. These associations indicate that ovarian cancers could respond to immunotherapy. However, immune checkpoint inhibitors (anti-CTLA-4 or anti-PD-1/PD-L1) have yielded modest clinical results in ovarian cancer patients [13,14]. A better understanding of the interplay between ovarian tumor cells and the immunological players in innate and adaptive immunity is critical for developing strategies to overcome the resistance of ovarian cancers to immunotherapy [15,16].

A major effector of innate immunity is the complement system, which represents one of the first lines of defense that distinguish “self” from “non-self” [17]. This system is composed of more than 50 soluble or membrane-bound effectors, regulators, and receptors, and it plays a relevant role in numerous physiological and pathological processes, including cancer [18]. Some evidence suggests that the modulation of complement activation may be exploited for the development of successful treatments against cancer [19,20]. In this review, we discuss the role played by components of adaptive and innate immunity on the development and progression of ovarian cancer. We mainly focus on the complement system, its role in the TME, and the rationale behind the use of complement modulators for the treatment of ovarian cancer.

## 2. Cellular and Humoral Immune Components of the Ovarian Tumor Microenvironment

The continuous feedback between tumor cells and the immune system is now recognized as a distinguished cancer hallmark [21]. Neoplastic transformation is characterized by the acquisition of tumor-associated molecular patterns that can be detected by the immune system. It is believed that upon recognition, innate and adaptive immunity can eliminate the vast majority of incipient cancer cells, avoiding tumor formation. However, the immune system is unable to eliminate all emerging malignant cells. When transforming cells escape from immune-mediated elimination, a dynamic interplay is established between tumor cells and the immune system, resulting in tumor-associated immune responses that may facilitate the development and progression of cancer [22]. In the case of ovarian tumors, a plethora of immune and non-immune cell types and non-cellular elements are found in the TME, not only in primary tumors but also in ascites and metastases [23]. The co-existence of multiple distinct tumor immune microenvironments within a single individual highlights the high plasticity and adaptability of ovarian cancers [24]. Herein, we summarize the main roles of the cellular and humoral elements of the immune system in ovarian tumor progression.

### 2.1. Cellular Immune Components

Tumor cells co-exist with non-immune and immune cells, and this relationship determines the natural history of the tumor and its resistance or response to therapy. The cellular immune components of the ovarian TME include T and B lymphocytes, natural killer (NK) cells, dendritic cells (DCs), polymorphonuclear cells, and macrophages.

T cells are a prominent component of the ovarian TME. Infiltration by CD8^+^ T cells is indicative of an ongoing immune response and is associated with a favorable prognosis [25]. Upon activation, tumor-specific CD8^+^ T cells secrete IFN-γ, tumor necrosis factor (TNF)-α, and cytotoxic mediators. However, in the ovarian TME, CD8^+^ T-cell responses are often dysfunctional. The autologous recognition of ovarian tumor antigens is limited to approximately 10% of the intratumoral CD8^+^ T receptor (TCR) repertoire [26]. This state can be attributed to the upregulation of T-cell exhaustion molecules by persistent antigen exposure and the existence of a hostile TME characterized by nutrient deprivation, hypoxia, oxidative stress, high concentrations of pro-inflammatory molecules, and the presence of immunosuppressive cell subsets [27]. In fact, ovarian cancers are highly enriched in Treg cells [28], a subset of lymphocytes that hamper tumor immunosurveillance by fostering peripheral tolerance to tumor antigens. Treg cells release and metabolize ATP to adenosine by the action of CD39 and CD73, a process that mediates immunosuppression via the adenosine and A_2A_ pathways [29]. Consequently, depletion of Treg cells in ovarian cancer-bearing mice effectively restores antitumor antigen-specific T-cell responses [30]. Other lymphoid subsets are important elements of the ovarian cancer immune infiltrate. In an orthotopic syngeneic mouse model, antitumor immunity was driven by CD4^+^ T cells [15]. A study identified a novel tumor-infiltrating NK subset characterized by a high expression of PD-1, reduced proliferative capability in response to cytokines, low degranulation, and impaired cytokine production upon interaction with tumor targets [31]. The presence of CD20^+^ B cells was associated with increased survival in ovarian cancer patients [32]. In human metastases of high-grade serous ovarian cancer, B cells develop memory responses in the TME and promote antitumor immune responses [33].

DCs are a diverse group of innate immune cells that infiltrate tumors and present tumor-derived antigens to naïve T cells. High densities of tumor-infiltrating DC-LAMP^+^ mature DCs suggest the establishment of an antitumor immune response, which is associated with a favorable prognosis in ovarian cancer patients [34]. However, this immune response is often rendered dysfunctional because of a variety of mechanisms, such as the upregulation of B7-H1 [35], the activation of the endoplasmic reticulum stress response factor X-box binding protein 1 (XBP1) [36], the attenuation of the toll-like receptor-mediated DC activation [37], and the activation of the cyclooxygenase 2 (COX2)/prostaglandin E_2_ (PGE_2_) axis to redirect the development of DCs toward the formation of myeloid-derived suppressor cells (MDSCs) [38].

MDSCs represent a heterogeneous population of immature myeloid cells that fail to differentiate into granulocytes, macrophages, or DCs. Two main subsets of MDSCs have been identified: polymorphonuclear MDSC (PMN-MDSC; CD11b^+^Ly6G^+^Ly6C^lo^ in mice and CD11b^+^CD14^−^CD15^+^CD66b^+^LOX-1^+^ in humans) and monocytic MDSC (M-MDSC; CD11b^+^Ly6G^−^Ly6C^hi^ in mice and CD14^+^CD15^−^HLA−DR^−/lo^ in humans). PMN-MDSCs and M-MDSCs are morphologically and phenotypically similar to neutrophils and monocytes, respectively [39]. These cells potently inhibit the anti-tumor immune response and reshape the TME to promote tumor growth and metastatic spread. The differentiation of myeloid precursors toward an MDSC phenotype is mediated by the inflammatory factor PGE_2_ via DNA methyltransferase 3A (DNMT3A)-dependent hypermethylation and the downregulation of a subset of myeloid genes [40]. The infiltration of MDSCs into ovarian tumors is associated with the Snail-mediated upregulation of CXCL1 and CXCL2 chemokines that attract MDSCs to the tumor via CXCR2 [41]. In the tumor niche, granulocyte–monocyte colony-stimulating factor (GM-CSF), through the signal transducer and activator of transcription 5 (STAT-5) pathway, upregulates AMP-activated protein kinase alpha 1 (AMPKα-1) in MDSCs to suppress antitumor CD8^+^ T-cell responses [42]. Both the presence of TNF-α and the production of NO by MDSCs sustain Th17 responses in the TME and myeloid cell recruitment in an IL-17-dependent manner [43,44]. 

Tumor-associated neutrophils, a cell population difficult to distinguish from PMN-MDSCs, are also involved in ovarian cancer-associated immune responses. In a KRAS-driven ovarian cancer mouse model, neutrophils reduced the amount of tumor-associated Treg cells and M-MDSCs while increasing the antitumor immune response via the upregulation of CD8^+^ T-cell function [45]. By contrast, the activation of neutrophils by mitochondrial DNA from ascites obstructs anti-tumor immunity and is associated with worse outcomes in patients with advanced ovarian cancer [46]. This study also reported the formation of neutrophil extracellular traps (NETs), networks of neutrophil decondensed chromatin fibers that are capable of binding tumor cells to support metastatic progression [47]. These contrasting roles of neutrophils in ovarian cancer have been attributed to different polarization states induced by the presence of transforming growth factor (TGF)-β and type-1 interferons in the TME [48].

Tumor-associated macrophages (TAMs) play a major role in the pathogenesis of ovarian cancer [49]. Macrophages constitute over 50% of the cells in peritoneal ovarian tumor nodules and malignant ascites and are involved in ovarian cancer initiation, progression, and metastasis [50]. TAMs are highly plastic cells that can exhibit two main phenotypes: anti-tumorigenic M1-like (F4/80^hi^ and CD86^+^ or CD80^+^ or iNOS^+^ in mice; CD68^+^HLA-DR^+^CD11c^−^ and CD86^+^ or CD80^+^ or iNOS^+^ in humans) and pro-tumorigenic M2-like (F4/80^hi^ and CD163^+^ or CD206^+^ or arginase^+^ in mice; CD68^+^HLA-DR^+^CD11c^−^ and CD163^+^ or CD206^+^ in humans). Analyses of TAM polarization in ovarian cancer show that M2 TAMs are associated with a poor prognosis [51,52]. Malignant cells direct TAM differentiation to facilitate tumor progression. The activation of the ovarian TAM pro-tumor phenotype requires the expression of zinc finger E-box binding homeobox 1 (ZEB1), a driver of the epithelial-mesenchymal transition (EMT), and involves direct crosstalk with tumor cells [53]. Tumor-expressed CD24 interacts with the inhibitory receptor sialic-acid-binding Ig-like lectin 10 (Siglec-10) expressed by ovarian cancer-inhibiting TAMs to avoid their antitumor effects [54]. Ovarian cancer cells skew co-cultured macrophages to a phenotype similar to that found in ovarian tumors [55]. Ovarian cancer cells promote membrane-cholesterol efflux and depletion of lipid rafts to polarize TAMs toward a tumor-promoting phenotype characterized by the upregulation of IL-4 signaling [56]. In return, TAMs enhance the malignant potential of ovarian cancer cells. Endothelial growth factor (EGF) secreted from TAMs promoted tumor growth at early stages of transcoelomic metastasis in a mouse model of ovarian cancer [57]. Moreover, TAMs enhance ovarian cancer invasiveness through activation of the nuclear factor kappa B (NF-kB) and Jun N-terminal kinase (JNK) pathways in tumor cells [58].

### 2.2. Humoral Immune Components

The crosstalk between the different cellular components of the TME is essential to reprogram tumor-associated immune responses. This process is orchestrated by complex networks interconnected by sets of soluble factors and extracellular structures, such as cytokines, chemokines, small metabolites, and microvesicles, among others [59]. In particular, cytokines mediate key interactions between immune and non-immune cells in the TME [60], and cytokine-based immunotherapy is a promising strategy to modulate the host’s immune response toward the induction of apoptosis in tumor cells [61]. To date, there are two FDA-approved treatments for melanoma and metastatic renal cell cancer based on the administration of TNF-α and interleukin (IL)-2 [62]. In the case of ovarian cancer, the proinflammatory cytokine IL-6 has been established as a key immunoregulator [63]. IL-6, along with other cytokines, activates pathways such as STAT and NF-kB, whose modulation could be used as a potential therapeutic tool [63].

Many years ago, Bjørge et al. found elevated levels of complement C1q, C3, C3a, and soluble C5b-9 in ascites from ovarian cancer patients, suggesting that local complement activation may constitute an important soluble component of the ovarian TME [64]. More recently, ovarian cancer has been classified as a cancer type with “upregulated complement” [65]. Interestingly, over the last decade, the complement system has emerged as a major non-cellular regulator of the TME in cancer immunity. Tumor-associated complement activation may support chronic inflammation, promote an immunosuppressive microenvironment, induce angiogenesis, and activate cancer-related signaling pathways [66]. In the case of ovarian cancer, complement dysregulation may even participate in the onset of tumors since complement molecules are already overexpressed in precursor lesions [67]. In the following section, we summarize the evidence supporting the involvement of the complement system in ovarian cancer progression.

## 3. The Complement System and Its Dual Role in Ovarian Cancer

**Figure 1:**
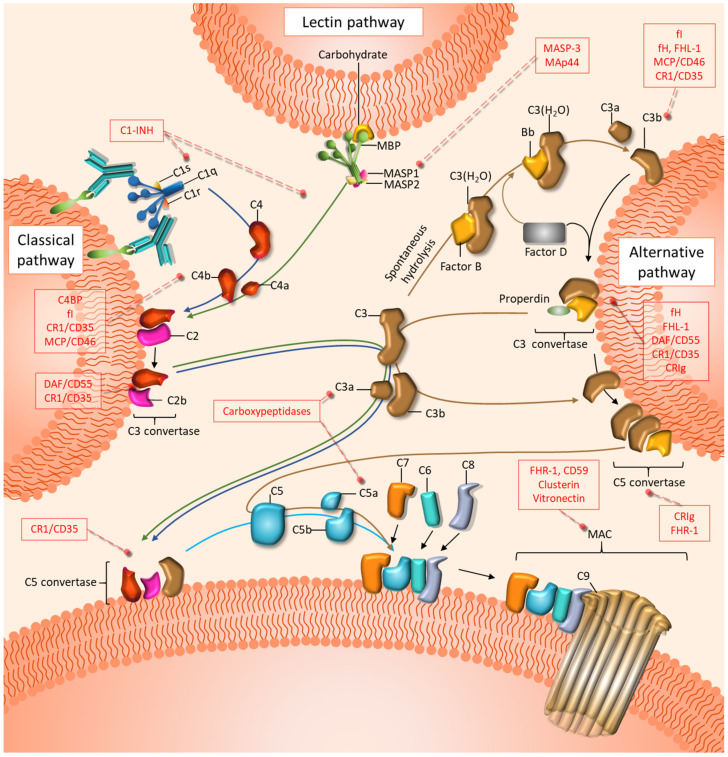
Schematic representation of the effectors and regulators of the complement cascade. Complement is initiated by three distinctive pathways: the classical (blue arrows), the lectin (green arrows), and the alternative (brown arrows) pathways. All three pathways converge in the formation of C3 and C5 convertases, which in turn generate the inflammation modulators C3a and C5a. The terminal steps, which culminate in the assembly of the membrane attack complex (MAC), are common to the three pathways. Inhibitory proteins of the three pathways are shown in red boxes.

In 1896, the complement system was first described as a heat-labile component in the serum able to “complement” heat-stable factors (antibodies). Now, the complement system is broadly known as a central part of the innate immune response composed of soluble and membrane-bound proteins that can coordinate a nonspecific inflammatory response against microbes and unwanted host elements [18]. Complement-circulating effectors are predominantly synthesized in the liver and are distributed throughout the body in an inactivated state. Complement can be activated by three main distinctive pathways: the classical pathway (CP), the lectin pathway (LP), and the alternative pathway (AP) (Figure 1). The three pathways converge in the cleavage of the complement component C3 into C3a and C3b. The CP is initiated in foreign, damaged, or dying cells when the C1 complex, which includes C1q, C1r, and C1s, recognizes antibody clusters, pathogen-associated molecular patterns (PAMPs), or danger-associated molecular patterns (DAMPs), among other molecules [68]. The LP is initiated by the recognition of carbohydrate patterns by mannose-binding lectin (MBL) or ficolins, along with the mannan-binding lectin serine proteases MASP1 and MASP2 [68]. The initiation of both the CP and the LP leads to the cleavage of C4 into C4a and C4b and, subsequently, C2 into C2a and C2b. The complex formed by C4b and C2b (C4bC2b, formerly C4b2a) constitutes the classical C3 convertase, which is responsible for the cleavage of C3 into C3a and C3b [68]. The AP is initiated by the spontaneous hydrolysis of C3 into C3(H_2_O), followed by its binding to factor B. This complex is recognized by factor D, which catalyzes the cleavage of factor B to form the fluid-phase alternative C3 convertase C3(H_2_O)Bb. This convertase can mediate the cleavage of C3 into C3a and C3b to form the membrane-bound alternative C3 convertase C3bBb [69]. Subsequently, C3b is able to bind to C4bC2b (in the CP and LP) or C3bBb (in the AP), leading to the formation of C5 convertase. This complex catalyzes the cleavage of C5 into C5a and C5b. The later fragment sequentially binds to C6, C7, C8, and C9 to form the cytolytic membrane attack complex (MAC) [18,68]. Many complement functions are mediated by the anaphylatoxins C3a and C5a, which act as potent inflammatory modulators [70]. These peptides signal through their respective G-protein-coupled receptors C3aR and C5aR1 [71]. A second, lesser-known C5a receptor, C5aR2, also participates in C5a responses, though its role remains unclear. Finally, an array of membrane and soluble complement regulatory proteins (CRPs) protects normal cells from the overactivation of complement [68] (Figure 1).

Complement plays an essential role in the control of cellular immunity [18], participating in the regulation, differentiation, and trafficking of several immune cell types [17,72]. C3 and C4 depletion impair humoral immune responses in vivo [73,74,75]. It has been postulated that antigen–antibody clusters interact with complement and are recognized by DCs, B lymphocytes, and macrophages [76]. Further evidence of the interplay between adaptive and innate immunity is the CD21(CR2)-CD19-CD81 complex on B cells, which enhances B-cell receptor function [77,78], partially by recognizing C3d-tagged surfaces [79]. Anaphylatoxins also play an important role in immune regulation. Most immune cell types express C3aR and/or C5aR1 on their surfaces [65]. On B cells, C3a impairs polyclonal immune responses and TNF-α and IL-6 production [80,81]. C5a has been extensively reported to induce the migration of several cell types [82,83,84,85,86]. Interestingly, C5a fosters antigen cross-presentation and the maturation of DCs [87,88,89]. Moreover, C3a-C3aR and C5a-C5aR1 signaling promote the activation [90] and expansion [91] of T cells and divert their differentiation from Treg cells [92,93]. Complement inhibitory proteins, such as CD46, have been shown to modulate T-cell fate depending on the isoform expressed and the presence of IL-2 [94,95]. Moreover, negative modulation of the inhibitor CD59 was demonstrated to ameliorate antigen-specific T-cell responses [96]. Overall, the information gathered during the past few decades illustrates the interconnections between the complement system and adaptive and innate immunity and endorses the hypothesis that complement’s role extends beyond its traditional non-specific, first-defense function.

Dysregulation of complement can lead to the development of several pathologies. Kidney diseases, such as atypical hemolytic uremic syndrome (aHUS) and C3 glomerulopathies, are closely related to complement anomalies. C3 glomerulopathies are characterized by the production of C3 fragments in the fluid phase via the alternative pathway and abnormal complement consumption that leads to the damage of the glomerular basement membrane [97]. Activation of the complement system is also involved in the pathogenesis of systemic autoimmune diseases [98]. Alterations in regulatory proteins can trigger serious conditions as well. Paroxysmal nocturnal hemoglobinuria (PNH) is a hematological disorder caused by a deficiency in glycosylphosphatidylinositol anchor synthesis that negatively affects the expression of the CRPs CD55 and CD59 [99]. More recently, cancer progression has been associated with complement activation [66].

In the next sections, we review studies that have reported the participation of components of the complement system in the biology of ovarian cancer or its potential clinical use. The findings of these studies are summarized in Table 1 and Table 2.

### 3.1. Complement Initiation Components in Ovarian Cancer

C1q, the first component of the classical complement activation pathway, links innate and adaptive immunity [123]. Both promoting and inhibitory roles have been reported for C1q in cancer progression, but most studies associate C1q expression with poor clinical outcomes in cancer, as is the case for gliomas and osteosarcomas [124,125]. C1q may act as a tumor-promoting factor through both complement-dependent and complement-independent mechanisms [126,127]. In ovarian cancer, the role of C1q appears to be context-dependent. In vitro, C1q displays an anti-tumor effect in SKOV3 cells by promoting apoptosis through the upregulation of the TNF-α pathway and the downregulation of the mammalian target of rapamycin (mTOR) survival pathway [100]. Conversely, expression levels of C1q in circulating extracellular vesicles isolated from ovarian cancer patients in stages III–IV are significantly elevated compared with those isolated from healthy individuals [115]. Discrepancies have also been observed in the case of the globular C1q receptor (gC1qR), a cell surface receptor for C1q. This molecule is upregulated in tumor cells [128], and its overexpression induces mitochondrial dysfunction and p53-dependent apoptosis in human cervical squamous carcinoma cells in vitro [101]. Consistently, the induction of gC1qR expression by paclitaxel in ovarian cancer cell lines SKOV3 and CAOV3 results in mitochondrial dysfunction and cell apoptosis [102]. However, this consistency observed in vitro disappears when clinical samples from ovarian cancer patients at different stages of the disease are analyzed. gC1qR downregulation was observed in ovarian cancer patients in the early stages of the disease (stages I–II) [102]. By contrast, gC1qR seems to be overexpressed in tumor tissue from ovarian cancer patients in stages III and IV, and this is associated with a poor prognosis and cisplatin resistance [116]. These data suggest an increase in complement activation during ovarian cancer progression. Consistent with this assumption, C4 was detected in ascitic fluid from late-stage patients, while it was undetectable in ascitic fluid from healthy donors [64]. Moreover, C4 levels were found to be upregulated in plasma samples from chemoresistant compared with chemosensitive ovarian cancer patients [119]. In the same study, complement factor I and C3 were found to be downregulated [119]. Finally, MBL and MASP2 serum levels are altered in ovarian cancer patients, and MBL levels are associated with advanced disease stages [117]. The ovarian tumor antigen cancer antigen 125 (CA-125), a highly glycosylated protein, may be a target for pattern recognition molecules, such as collectins and ficolins, which may mediate the interaction with MBL and the activation of the lectin pathway [129]. Serum ficolins have been reported to be elevated in ovarian cancer patients despite their lower tumor expression [118]. In conclusion, several studies have reported the presence of complement initiation factors in ovarian cancer. However, the contribution of these factors to ovarian cancer progression and response to treatment is still unclear and requires further investigation. 

### 3.2. C3 and C5 in Ovarian Cancer

The C3- and C5-derived fragments C3a and C5a participate in the establishment of a chronic inflammatory state that may favor tumorigenesis and cancer progression [70]. In ovarian cancer, the implication of C3a and C5a seems to depend on multiple factors, although most of the evidence suggests a tumor-promoting effect. Nuñez-Cruz et al. assessed the role of complement in ovarian tumor progression using C3 and C5aR1-deficient mice. Complement inhibition impaired both tumor vascularization and growth [103]. Some molecular mechanisms have been associated with the tumor-promoting function of C3 and C5 in ovarian cancer tumor cells. These mechanisms include the activation of the phosphatidylinositol-3-kinase (PI3K) pathway and the induction of EMT [104,105]. C3 and C5 and their effector fragments also influence tumor progression by acting on immune cells. Circulating polymorphonuclear cells from ovarian cancer patients can acquire an immunosuppressive phenotype capable of restraining T-cell proliferation after exposure to ascites in a process dependent on C3 [130]. This T-cell non-responsiveness is associated with the production of C5a and is mediated by mTOR signaling and nuclear factor of activated T-cells (NFAT) translocation [131]. Interestingly, C5a may function in a dose-dependent manner. Thus, in a SKOV-3 tumor model, low local doses of C5a reduced tumor growth in association with the recruitment of M1 TAMs and NK cells, while high doses promoted tumor progression [107]. Ovarian cancer cells overexpress ribosomal protein S19 (RPS19), which leads to tumor growth through its interaction with C5aR1 in MDSCs [132]. By contrast, the local production of C3 and the release of C5a disrupt the tumor endothelial barrier, facilitating the homing of T cells and their tumor recruitment [106]. This study further stresses the contrasting effects associated with complement effectors in different models of ovarian cancer. Unfortunately, the results reported in patients do not clarify the matter. High levels of C3 or C5aR1 have been associated with decreased overall survival [104,133]. By contrast, reduced expression of C3 was observed in the blood of ovarian cancer patients [134], and this factor was downregulated in the serum of platinum-resistant patients [119].

### 3.3. Complement Regulatory Proteins in Ovarian Cancer

CRPs protect host cells from autologous complement attack, but they can render complement ineffective at eliminating cancer cells. Membrane-bound CRPs (mCRPs), such as CD46, CD55, and CD59, are expressed by ovarian cancer tumors [121,135] and cell lines [108,122,135]. These regulators are linked to worse clinical outcomes and may constitute an obstacle for cancer immunotherapy [121,136,137,138]. Their presence has also been associated with the development of multi-drug resistance in ovarian cancer cells [139]. Neutralization of mCRPs increases the sensitivity to complement-dependent cytotoxicity [111,113,139], reduces ovarian tumor growth [110], and enhances the anti-tumor efficacy of therapeutic antibodies [108,112]. In line with these findings, CD55 silencing restores cisplatin sensitivity to chemotherapy in resistant ovarian cancer cells [109]. Regarding soluble complement regulators, a range of studies has demonstrated their importance in several tumor types [140,141,142,143]. In ovarian cancer, some soluble complement inhibitors, such as factor H and factor H-like 1 (FHL-1), have been found in ascitic fluid and primary tumors [64,114]. However, the role of these regulators in ovarian cancer progression has not been defined yet. 

**Figure 2:**
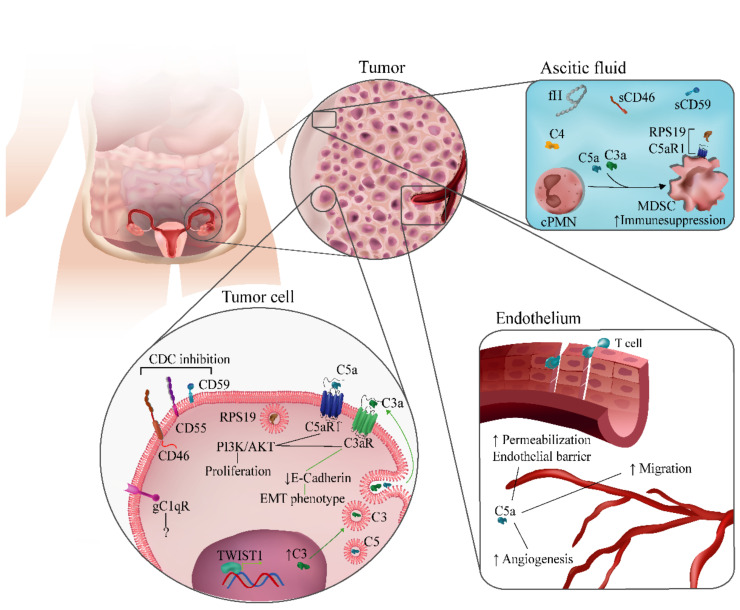
Complement-related mechanisms associated with ovarian cancer progression. Complement components have been implicated in different biological processes associated with ovarian cancer progression. They include modulation of immunosuppression in the tumor microenvironment; regulation of angiogenesis and endothelial permeabilization; autocrine and paracrine effects in tumor cells mediated by C1q, C3a, or C5a; and inhibition of complement-dependent cytotoxicity (CDC) by membrane-bound complement regulators.

In conclusion, the evidence suggests that complement dysregulation drives ovarian cancer progression. Complement effectors, receptors, and regulators have been implicated in different aspects of ovarian cancer biology (Figure 2). Although there are inconsistencies in the description of the role of complement components in some clinical or experimental contexts, the majority of studies point toward a tumor-promoting activity of complement in well-established tumors. These findings have paved the way for studies aimed to potentiate cancer therapies through the modulation of the complement system.

## 4. Therapeutic Potential of Targeting Complement in Ovarian Cancer

Complement inhibition may be a useful therapeutic strategy against cancer [19]. Agonists of C5aR1 and C3aR increase ovarian tumor cell proliferation, migration, and invasion, suggesting that receptor antagonists could be used to block cancer growth [104]. Complement targeting may also impair angiogenesis, a highly relevant biological process in ovarian cancer. Elevated levels of serum VEGF after chemotherapy treatment have been associated with lower overall survival in ovarian cancer patients [144], and the anti-VEGF antibody bevacizumab has shown therapeutic activity in both patients and animal models [145,146,147]. Genetic or pharmacological inhibition of C3 or C5aR1 results in smaller and poorly vascularized ovarian tumors in vivo [103], and C5a is able to promote endothelial cell tube formation and migration [103,148]. Therefore, it can be speculated that inhibition of complement may potentiate the efficacy of anti-angiogenic agents.

Another scenario in which complement modulation may be of special relevance is immunotherapy. We previously described the implication of effectors and regulators of the complement system in the ability of T cells to infiltrate tumors and the response against tumor-associated antigens [149]. Using various models of lung cancer, we proposed that the modulation of complement activation can improve the antitumor efficacy of monoclonal antibodies targeting the PD-1/PD-L1 pathway [150]. This synergistic effect has also been reported in other tumor models targeting C5a/C5aR1 [151,152] or C3a/C3aR [151,153]. To our knowledge, these combinations have not been tested yet in models of ovarian cancer, and we can only hypothesize about the outcome of these studies. The inhibition of C3 or C5aR1 abrogates the suppressor phenotype of MDSCs in the ovarian TME [130,131], suggesting that complement inhibition may have a positive effect on the efficacy of anti-PD-1/PD-L1 therapies. Conversely, antitumor T cells require the production of C3 and the release of C5a in the endothelium in order to infiltrate ovarian tumors [106]. The targeting of mCRPs should also be considered in light of their relevance in the TME [136]. The inhibition of mCRPs may be used to sensitize tumors to other drugs. In ovarian cancer, the neutralization of CD46, CD55, and CD59 in combination with the anti-HER2 monoclonal antibodies trastuzumab and pertuzumab induces tumor cell killing in vitro [113]. Nevertheless, considering the dual role of complement molecules in ovarian tumors, in vivo studies are needed to determine whether complement inhibition has any impact on the response to checkpoint-based or antibody-based immunotherapies, and in which direction.

## 5. The Need for Preclinical Models to Better Delineate the Role of Complement in Ovarian Cancer

In this review, we discussed the functions that complement components exert in the biology of ovarian tumors. Many questions remain regarding the conflicting results observed in different experimental settings. To address these questions, in vivo models that faithfully recapitulate the complexity of the disease are needed. Currently, there are a few animal models established for the study of ovarian cancer. These include genetically engineered mouse models, xenograft cell transplants of human cell lines, and patient-derived xenografts [154]. These models have facilitated the study of many mechanisms associated with ovarian cancer progression and have allowed the evaluation of many therapeutic molecules [155]. For the study of complement-related mechanisms or treatments, mouse models that capture the complexity of the TME are required. Models based on syngeneic tumor cells injected intraperitoneally in immunocompetent mice represent a practical option. Some studies have used the syngeneic intraperitoneal injection of ID-8-MOSEC, a mouse epithelial ovarian cancer cell line originating in C57BL/6 mice, to evaluate the roles of C3, C5, and C5aR1 in ovarian cancer development and progression (Table 1) [104,106]. This cell line was developed by Dr. Katherine F. Roby in the Department of Anatomy and Cell Biology of the University of Kansas in the early 2000s, and it is one of the most frequently used ovarian cancer cell lines since it has the capacity to induce tumor peritoneal implants observed in stages III and IV [156]. Because of its slow growth rate, some strategies have been developed to increase the aggressiveness of this cell line, including the overexpression of dendritic cell chemoattractant beta-defensin 29 (Defb29) or VEGF [157], two factors associated with increased invasiveness. Nevertheless, this model does not completely recapitulate the human pathophysiology of the disease and does not exactly reproduce the TME [154]. The development of better ovarian cancer models is needed to unravel the mechanisms by which complement components modulate ovarian cancer progression and to evaluate complement-based therapeutic combinations.

## 6. Conclusions

A growing body of literature suggests that the complement system is involved in ovarian cancer progression. Nevertheless, the specific role of the different complement components in different clinical scenarios has just started to be unraveled, and many answers remain elusive. The molecular heterogeneity of ovarian cancers and the complexity of the biological interactions in the ovarian TME pose a challenge to our understanding of the mechanisms underlying the complement-associated immune responses and the identification of adequate therapeutic targets. The situation is aggravated by the lack of preclinical models that reliably recreate ovarian cancer traits. Therefore, further studies are needed to better delineate the complement-related mechanisms associated with ovarian cancer progression as well as to determine how complement activation should be modulated to treat ovarian cancer patients.

## Figures and Tables

**Table 1:** Summary of the studies in ovarian cancer cell lines and mouse models reporting tumor-promoting or tumor-suppressing activities mediated by complement components.

Component Type	Complement Component (s)	Role in Cancer	Experimental Setting	Cell Line(s)	In Vivo Model	Mechanism	Ref
Complement effectors and receptors	C1q	Anti-tumor	In vitro	SKOV3	-	Induction of apoptosis	[100]
	gC1qR	Anti-tumor	In vitro	C33a, SiHa	-	Induction of apoptosis	[101]
	gC1qR	Anti-tumor	In vitro	SKOV3, CAOV-3	-	Induction of apoptosis after paclitaxel treatment	[102]
	C3 and C5aR1	Pro-tumor	In vivo	-	Spontaneous model in C57BL/6 TgMISIIR-Tag mice	Inhibition of angiogenesis	[103]
	C3aR and C5aR1	Pro-tumor	In vivo	ID-8 VEGF	Syngenic model in C57BL/6 mice	Autocrine stimulation of tumor growth	[104]
	C3	Pro-tumor	In vivo	ID-8 VEGF	Syngenic model in C57BL/6 mice	Autocrine promotion of EMT	[105]
	C3 and C5aR1	Anti-tumor	In vivo	TC-1	Syngenic model in B6.SJL-PtprcaPep3b/BoyJ mice	Promotion of T-cell homing	[106]
	C5a	Anti-tumor Pro-tumor	In vivo	SKOV-3	Xenograft model in SCID mice	Dose-dependent effect on tumor growth	[107]
Complement regulators	CD59, CD46, FH, and FHL-1	Pro-tumor	In vitro	Caov-3, SK-OV-3, SW626, PA-1, HUV-EC-C	-	Functional complement activation and regulation occurs locally in ascites	[64]
	CD55	Pro-tumor	In vivo	SK-OV-3	Xenograft model in SCID* mice	Blockade of CD55 leads to improved efficacy of mAb therapy	[108]
	CD55	Pro-tumor	In vivo	A2780, TOV112, CP70, HEC1a	Xenograft model in SCID mice	Silencing of CD55 restores sensitivity to chemotherapy	[109]
	CD59	Pro-tumor	In vivo	A2780	Xenograft model in SCID mice	Silencing of CD59 reduces tumor growth	[110]
	CD59	Pro-tumor	In vitro	SK-OV-3	-	Neutralization improves CDC mediated by mAb therapy	[111]
	CD46 and CD59	Pro-tumor	In vitro	IGROV1, OVCAR3, SKOV3, OAW42, INTOV1, INTOV2	-	Neutralization improves CDC mediated by mAb therapy	[112]
	CD46, CD55, and CD59	Pro-tumor	In vitro	SK-OV-3	-	Silencing of CRPs leads to improved efficacy of mAbs	[113]
	FH, FHL-1, and sCD46	Pro-tumor	In vitro	SK-OV-3, Caov-3, PA-1, SW626	-	Resistance to CDC	[114]

EMT: epithelial-mesenchymal transition, SCID: severe combined immunodeficient, mAb: monoclonal antibody, CDC: complement-dependent cytotoxicity.

**Table 2:** Summary of the studies performed with clinical samples reporting the potential clinical use of the determination of complement components.

Component Type	Complement Component(s)	Role in Cancer	Type of Sample	Methodology	Stage(s)	Mechanism	Ref
Complement effectors and receptors	C1q	Diagnosis	Serum	Mass spectrometry	III–IV	Overexpression	[115]
	gC1qR	Prognosis	Tissue	IHC	III–IV	Overexpression associated with shorter overall survival	[116]
	MBL and MASP-2	Diagnosis	Serum	ELISA	I–IV	Overexpression	[117]
	Ficolin-2 and ficolin-3	Diagnosis	Serum	ELISA	I–IV	Overexpression	[118]
	C3 and C4	Prediction of response	Plasma	Mass spectrometry	III–IV	Downregulation (C3) or upregulation (C4) in platinum-resistant patients	[119]
	C3	Diagnosis	Serum	Mass spectrometry	I–IV	Downregulation	[120]
	C3 and C5aR1	Prognosis	Tissue	Real-time PCR	I–II	mRNA levels associated with decreased overall survival	[104]
Complement regulators	CD59, CD46, FH, and FHL-1	Pro-tumor	Ascitic fluid	Immunoblotting, ELISA, IHC	I, III, IV	Complement activation and regulation occurs locally in ascites	[64]
	CD46	Prognosis	Tissue	IHC	I–III	Expression associated with shorter survival	[121]
	CD46 and CD59	Therapy	Tissue	cDNA microarray, IHC	Advanced stage	Neutralization improves CDC mediated by mAb therapy	[112]
	CD46, CD55, and CD59	Pro-tumor	Tissue	IHC	Not specified	Overexpression in malignant tissue	[122]
	FH, FHL-1, and sCD46	Pro-tumor	Ascitic fluid, tissue	ELISA, IHC	III–IV	Overexpression in malignant tissue	[114]

IHC: immunohistochemistry, mAb: monoclonal antibody, CDC: complement-dependent cytotoxicity.
